# Floral Integration and Phenotypic Selection on Floral Traits of *Ipomoea cavalcantei* (Convolvulaceae), a Rare, Endemic, and Endangered Species to the Amazon Ironstone Outcrops

**DOI:** 10.1002/ece3.72133

**Published:** 2025-09-13

**Authors:** Adriano Valentin‐Silva, Ana Carolina Galindo da Costa, Arthur Domingos‐Melo, Lucas Erickson Nascimento da Costa, Kleber Resende Silva, Carolina da Silva Carvalho, Maurício Takashi Coutinho Watanabe

**Affiliations:** ^1^ Instituto Tecnológico Vale Belém Brazil; ^2^ Instituto de Estudos Do Xingu Universidade Federal Do Sul e Sudeste Do Pará São Félix do Xingu Brazil; ^3^ Laboratório de Biologia Floral e Ecologia Reprodutiva Universidade Federal de Sergipe—Campus Prof. Alberto Carvalho Itabaiana Brazil

**Keywords:** canga, evolutionary potential, floral morphometry, mating system, morning glories

## Abstract

Maintaining evolutionary potential is essential for species persistence, particularly for rare and endemic taxa that are more susceptible to habitat changes. We evaluated floral integration and phenotypic selection on the floral traits of *Ipomoea cavalcantei*, a species endemic to the ironstone outcrops (*cangas*) of the Eastern Amazon and primarily pollinated by hummingbirds. Using 90 individuals from a natural population in the Floresta Nacional de Carajás, we conducted six pollination experiments to assess the mating system. We estimated individual fitness through fruit set and characterized floral phenotype by measuring ten traits under a stereomicroscope. We analyzed the mating system using a generalized linear model, assessed floral integration through matrix correlations, and evaluated phenotypic selection via simple and multiple regressions. *Ipomoea cavalcantei* was found to be self‐incompatible, with higher fruit set in pollen‐supplemented flowers. The association between matrices (modularity) was significant for both the morphological and developmental criteria, and style length exhibited five significant correlations out of the nine. Contrary to expectations for hummingbird‐pollinated flowers, corolla‐associated traits were not under significant selection. Only the ovary width showed a significant selection differential and gradient in the linear analyses, likely due to selective pressure from pre‐dispersal seed predators. This indicates positive directional selection: individuals with flowers bearing wider ovaries than the population mean tend to have higher relative fitness. Our findings highlight the role of pollinators in shaping floral integration in *I. cavalcantei* and their importance in ensuring maximum seed production, thereby supporting offspring formation even under seed predation.

## Graphical abstract

This paper analyzed the evolutionary potential of *Ipomoea cavalcantei* (an endemic species from the Amazon) by using floral integration and phenotypic selection analysis. The analyzed population presented floral integration in style length, probably due to selective pressures from pollinators on floral traits related to pollen deposition. Furthermore, we observed that *I. cavalcantei* flowers have variation in ovary width, with positive directional selection, an uncommon result in phenotypic selection studies.

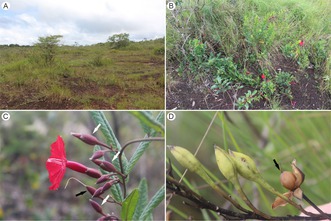

## 1 Introduction

We are experiencing a biodiversity crisis with widespread species loss driven by anthropogenic environmental change (Kindsvater et al. 2018), which calls for strategies to reduce extinction risk (Yang et al. 2020). Maintaining evolutionary potential, that is, the capacity to evolve traits that enhance population fitness in response to change, can support species persistence under current environmental pressures (Forester et al. 2022). This involves conserving adaptive diversity and preserving the processes that shape evolutionary change (Forester et al. 2022). The situation is more critical for endemic and rare species, which are more susceptible to habitat changes (Murray et al. 2002; Wright and Muller‐Landau 2006). These species may also experience pollination limitations, particularly when they depend on biotic vectors for sexual reproduction (Alonso et al. 2010, 2013), which can constrain their evolutionary potential.

In angiosperms, pollinator‐mediated selection contributes to evolutionary change (Strauss and Whittall 2006; Van der Niet et al. 2014). Floral morphology varies at multiple scales (intraindividual and intrapopulation), and selection can act on the entire flower or on specific floral modules (Berg 1960; Ordano et al. 2008). Phenotypic variation occurs in both specialist and generalist pollination systems (Galetto et al. 2023). Although pollinators often mediate this process, other ecological interactions can also influence natural selection (Strauss and Whittall 2006). These factors may act independently, interact with pollinators, or oppose pollinator‐driven selection (Waser and Campbell 2004; Strauss and Whittall 2006). Among the traits under selection are those linked to pollinator attraction and pollination efficiency (Sletvold 2019), which can affect reproductive success. This pattern has been observed in specialized pollination systems (Van der Niet and Johnson 2012; Barrett 2013). In generalist pollination systems, floral traits may reflect adaptation to different functional groups of pollinators or may exhibit traits that are not specifically adapted to any particular pollinator group (Gómez, Muñoz‐Pajares, et al. 2014; Valentin‐Silva et al. 2021).

Selection of floral traits can result in phenotypic integration, defined as covariation among traits arising from historical, physiological, developmental, and adaptive factors (Armbruster et al. 2004). Floral integration is considered an adaptation that enhances morphological fit between flowers and pollinators, which can improve pollination efficiency and influence reproductive success (Berg 1960; Armbruster et al. 2004). The level of integration may vary with pollination and mating systems and is reported to be greater in specialist and self‐compatible species (Rosas‐Guerrero et al. 2011; Fornoni et al. 2016; Dellinger et al. 2019). Within flowers, integration tends to be stronger at the intrafloral scale than across the whole structure, highlighting the role of modularity in plant adaptation (Ordano et al. 2008; Dellinger et al. 2019).

*Ipomoea* is the largest genus in Convolvulaceae, with at least 650 species (Eserman et al. 2020). Floral size varies among individuals in several species (Bullock et al. 1987; Murcia 1990; Parra‐Tabla and Bullock 2003; McCallum and Chang 2016). Most *Ipomoea* species are pollinated by bees (Real 1981; Stucky and Beckmann 1982; Bullock et al. 1987; Devall and Thien 1989; Galetto et al. 2002; Kiill and Ranga 2003; Pacheco Filho et al. 2011), but visits by butterflies and hummingbirds have also been recorded (Galetto and Bernardello 2004; Wolfe and Sowell 2006). In species with nocturnal floral anthesis, bats and moths act as pollinators (McMullen 2009; Raju et al. 2011; Domingos‐Melo et al. 2023). Some species are self‐compatible (Delgado‐Dávila et al. 2016) and thus may not depend entirely on pollinators, which could reduce the strength of selection exerted by them.

*Ipomoea cavalcantei* D.F.Austin is endemic to the ironstone outcrops (*cangas*) of the Eastern Amazon in Serra dos Carajás and is listed as threatened with extinction (Simão‐Bianchini et al. 2016; Giulietti et al. 2019; Rodrigues et al. 2020). Its flowers are phenotypically specialized (*sensu* Ollerton et al. 2007), with traits consistent with ornithophily, such as a bright red hypocrateriform corolla and exserted reproductive organs (Babiychuk et al. 2017), and show variation among individuals (Babiychuk et al. 2019). Floral divergence between *I. cavalcantei* and its sister species *I. marabaensis* D.F.Austin & Secco, which is melittophilous and also occurs in Serra dos Carajás, suggests pollinator‐mediated adaptive radiation (Babiychuk et al. 2017), indicating a role for pollinators in floral diversification in the genus. *Ipomoea cavalcantei* is visited by at least nine hummingbird species, along with long‐tongued bees (two *Eulaema* spp.) and nectar robbers (Babiychuk et al. 2019), characterizing an ecologically (number of pollinators) and functionally (taxonomic diversity of pollinators) generalist pollination system (*sensu* Ollerton et al. 2007).

Given its conservation status, floral variation among individuals, and a potentially mixed pollination system, *I. cavalcantei* offers a suitable case for examining trait associations and current selection in a natural population. We assessed floral trait variation and characterized the mating system to evaluate floral integration and phenotypic selection. Specifically, we asked: (1) How does floral phenotype vary, and which traits exhibit floral integration? (2) Does the species depend on biotic pollinators for sexual reproduction? (3) Which floral traits are under selection in the population? We hypothesized that *I. cavalcantei* shows floral integration and that some traits are under positive selection related to biotic pollinators. We expected our results to align with existing hypotheses on floral integration (Rosas‐Guerrero et al. 2011; Fornoni et al. 2016; Dellinger et al. 2019) and phenotypic selection (Van der Niet and Johnson 2012; Barrett 2013; Gómez, Muñoz‐Pajares, et al. 2014; Valentin‐Silva et al. 2021) based on pollination and mating systems.

## 2 Materials and Methods

### 2.1 Study Area and Species

We conducted the study in the Floresta Nacional (FLONA) de Carajás, which comprises ironstone outcrops (*canga*) surrounded by dense or open rainforest vegetation (IBAMA 2003; Schaefer et al. 2016). The FLONA de Carajás is a protected area for sustainable use of natural resources, located in the eastern Brazilian Amazon, covering 411,949 ha and with elevations from 150 to 897 m a.s.l. (IBAMA 2003). According to the Köppen system, the regional climate is Aw (humid tropical, with a dry winter and precipitation in the driest month < 60 mm; Alvares et al. 2013). Mean annual precipitation is 1801 mm, and mean annual temperature is 27.4°C (meteorological data for 2001–2021) (Instituto Tecnológico Vale, unpublished data).

**FIGURE 1:**
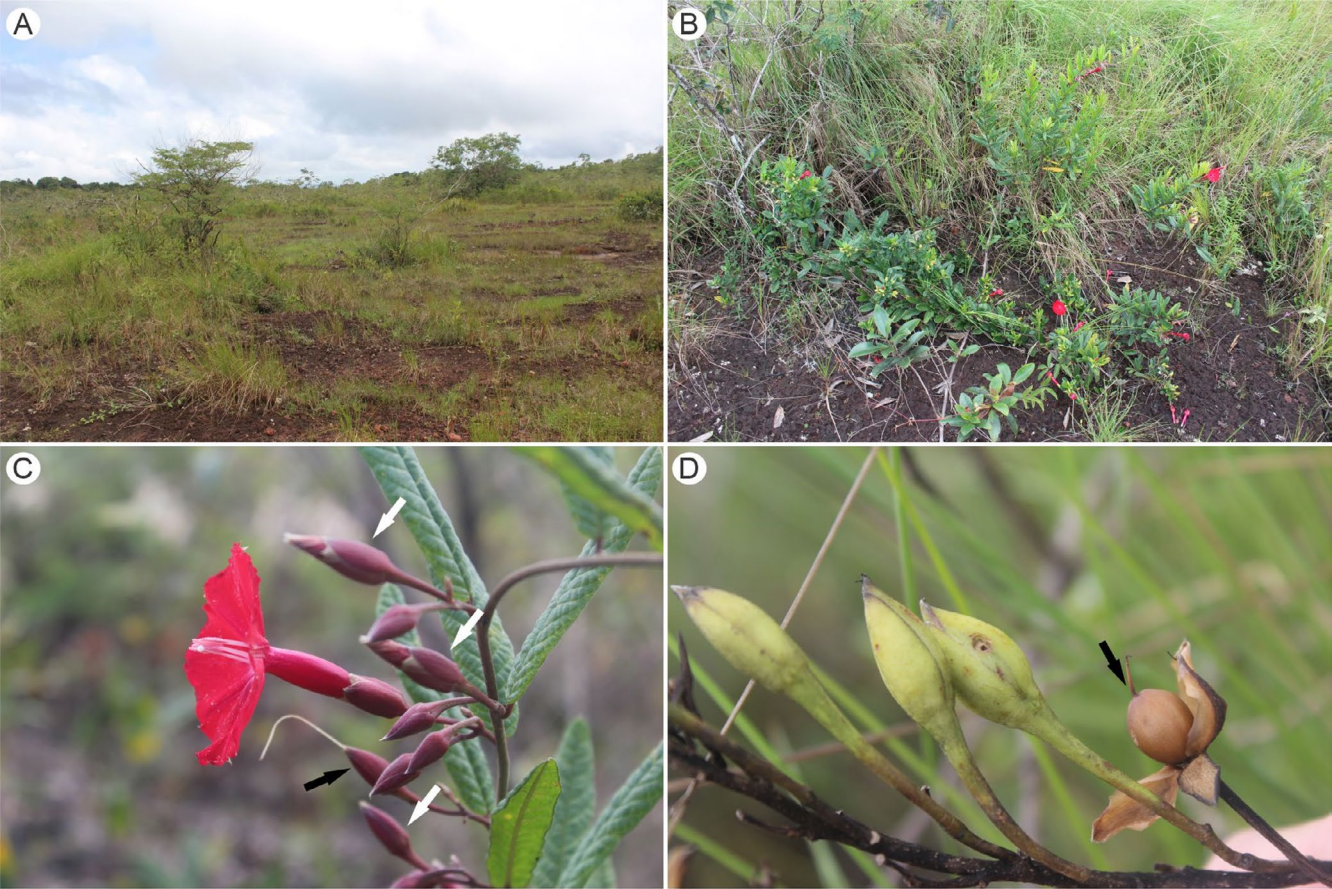
Characterization of *Ipomoea cavalcantei*. (A) Natural habitat. (B) Reproductive individual. (C) Floral buds (white arrows) and flowers at anthesis and post‐anthesis (black arrow). (D) Immature and mature fruits (arrow).

*Ipomoea cavalcantei* is a liana or shrub with entire, oblong to obovate leaves, cymose inflorescences with 1 to 3 flowers, bright red hypocrateriform corollas with epipetalous stamens, capsule‐type fruits, and seeds with trichomes on the sides (Simão‐Bianchini et al. 2016; Figure 1). The species is restricted to Serra Norte in FLONA de Carajás and is classified as a highly restricted endemic (Giulietti et al. 2019). Flowering occurs from January to July, and fruiting occurs from April to July (Costa et al. 2023).

### 2.2 Data Collection

**FIGURE 2:**
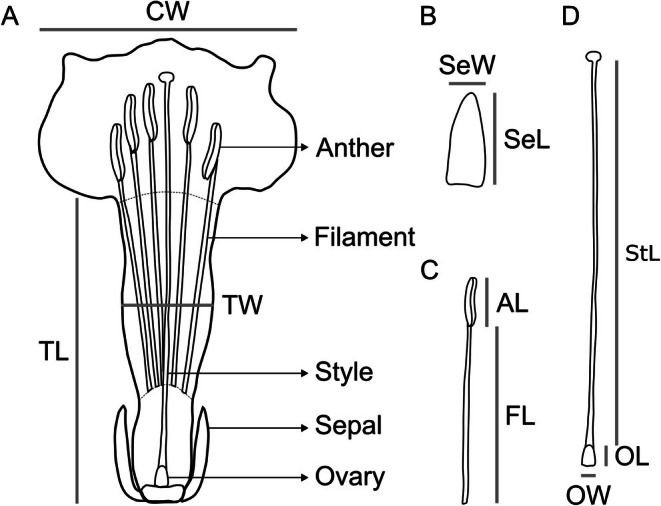
Floral traits measured in the morphometric analysis of *Ipomoea cavalcantei*. (A) Flower showing corolla traits. (B) Calyx traits. (C) Androecium traits. (D) Gynoecium traits. AL = anther length; CW = corolla width; FL = filament length; OL = ovary length; OW = ovary width; SeL = sepal length; SeW = sepal width; StL = style length; TL = corolla tube length; TW = corolla tube width.

We tagged 90 individuals of *I. cavalcantei* on Serra Norte (N1 plateau; 6°1′41″ S, 50°16′37″ W) to evaluate floral integration and phenotypic selection. This population is located in an area without iron mining activity. To characterize the floral phenotype, we collected one to three flowers per plant and preserved them in 70% ethanol for laboratory analysis. Flowers were dissected under a stereomicroscope (Zeiss, SteREO Discovery V12) equipped with a camera (Zeiss, AxioCam 712 color), and morphometric measurements were taken using ZEN 3.4 (blue edition). We measured the following traits (one to three flowers per trait per individual): sepal length and width, corolla width, corolla tube length and width, filament and anther length, ovary length and width, and style length (Figure 2). Given the presence of floral visitors from different functional groups, including pollinators and nectar robbers (Babiychuk et al. 2019), we included all measured traits in subsequent analyses to account for potential selective pressures from these agents (Waser and Campbell 2004; Strauss and Whittall 2006).

We characterized the mating system and estimated fitness using the same individuals sampled for floral morphometry. To determine the mating system at the population level, we conducted six pollination treatments (Richards 1997; Sage et al. 2005) between February and April 2022. (1) Spontaneous self‐pollination: pre‐anthesis flower buds were isolated with fabric bags to exclude pollinators (*N* = 90, one flower per individual). (2) Manual self‐pollination: bagged flowers were self‐pollinated during anthesis using their own pollen (*N* = 90, one flower per individual). (3) Geitonogamous pollination: flower buds were emasculated, bagged, and pollinated with pollen from other flowers of the same individual (*N* = 90, one flower per individual). (4) Cross‐pollination: emasculated, bagged flower buds were pollinated with pollen from different individuals (*N* = 90, one flower per individual). (5) Pollen supplementation: manual outcross pollen was applied in intact flowers without bagging (*N* = 191, one to three flowers per individual). (6) Natural pollination (control): pre‐anthesis buds were marked and left exposed during anthesis (*N* = 178, one to two flowers per individual). Because flowers are short‐lived (Babiychuk et al. 2019), all pollinations were performed in the morning using fresh pollen. In treatments 1 to 4, flowers/fruits remained bagged for approximately 30 days. Manual pollinations (treatments 2 to 5) were conducted with wooden toothpicks, which were replaced for each flower to prevent cross‐contamination. For treatments 4 and 5, pollen donors were selected from individuals with pre‐bagged flowers to prevent pollen loss. Depending on distance and flower availability, different individuals were used as pollen sources.

We also assessed fitness at the individual level (*N* = 89) by counting flowers and fruits on two branches per plant. In both approaches, seed number per treatment was quantified using immature fruits to avoid losses due to dehiscence and seed predation (Santos et al. 2023). At this stage, developing seeds were distinguishable from ovules by size and the presence of trichomes, which occur only on seeds.

### 2.3 Statistical Analysis

We calculated the coefficient of variation (CV) for each floral trait and performed principal component analysis (PCA) and variance partitioning to describe phenotypic variation. Prior to analysis, we retained only individuals with both morphometric data from at least one flower and fitness data (*N* = 81). For CV calculations, we used the mean of each floral trait per individual, standardized (mean = 0, SD = 1), to assess intrapopulation variation. Confidence intervals (95% CIs) for CVs were estimated using McKay's approximation (McKay 1932). For traits with CV > 33%, we applied a noncentral t‐distribution, following general recommendations (Smithson 2003). Traits were considered uniform when both the point estimate and 95% CI of the CV were ≤ 10% (Zhou et al. 2021). In PCA and variance partitioning, individuals with only one measured flower were excluded; we used all flower‐level measurements (*N* = 66, two to three flowers per individual) to capture intra‐individual variation. PCA was conducted on standardized data.

Floral integration was assessed using three approaches. First, we used the empirical correlation matrix among floral traits (*N* = 81) to identify the most integrated floral parts (Domingos‐Melo et al. 2018). To evaluate the precision of the correlations, we generated 95% CIs using bootstrap resampling (9999 iterations; Davison and Hinkley 1997). Second, we tested for the presence of phenotypic modules by comparing the empirical matrix with three hypothetical correlation matrices, following the method proposed by Cheverud et al. (1989). In these matrices, a value of 1 was assigned to trait pairs expected to be correlated and 0 to those not expected to be correlated (Baranzelli et al. 2014). The modules were defined based on field observations. The morphological criterion (matrix 1; Table S1) considers each floral whorl (calyx, corolla, androecium, and gynoecium) an independent unit, with high correlations expected within but not between whorls. The functional criterion (matrix 2; Table S2) groups traits by floral function, forming three modules: (1) visual attraction (calyx and corolla), considering the color variation in sepals (green to vinaceous); (2) pollen export (androecium); and (3) pollen reception (gynoecium). The developmental criterion (matrix 3; Table S3) incorporates flower ontogeny and fusion patterns, assigning high correlations among traits from sepals, epipetalous stamens, and pistil. We compared each empirical and hypothetical matrix pair using Mantel tests (Pearson correlation coefficient, 999 permutations). Confidence intervals (95%) for the Mantel coefficients were estimated through bootstrap resampling (1000 iterations), using the 2.5th and 97.5th percentiles of the resulting distribution (Davison and Hinkley 1997). Correlation strength was categorized as weak (*r* < 0.3), moderate (0.3 < *r* < 0.5), or strong (*r* ≥ 0.5) (Cohen 1988). Finally, we calculated the Wagner–Cheverud index (*I*_*c*_) to quantify the degree of phenotypic integration, based on the correlation matrix of floral traits (Wagner 1984; Cheverud et al. 1989). High *I*_*c*_ values indicate that few eigenvalues explain most of the variance (strong integration), whereas low values reflect a more even variance distribution across eigenvalues (weak integration).

We evaluated the mating system based on fruit set in the pollination experiments (*N* = 90). Because each fruit consistently contained four seeds, seed count per fruit was not used in the analysis. A generalized linear model (GLM) with a binomial error distribution and complementary log–log link function was fitted. The number of fruits produced and pollination experiments were considered as response and explanatory variables, respectively. Due to underdispersion (residual deviance/df = 0.76), a quasibinomial distribution was used. The best model was selected using likelihood ratio tests (LRT; Zuur et al. 2009). *Post hoc* Tukey tests were applied to evaluate treatment differences.

Phenotypic selection was examined following Lande and Arnold (1983), using linear and quadratic regressions of fitness on phenotype to estimate the form and strength of selection (Palacio et al. 2019). This method captures linear (directional), nonlinear (stabilizing or disruptive), and correlational (interaction) selection. The analysis included 81 individuals with both traits (mean values of each floral trait per individual) and fitness data. Phenotypic traits were standardized (mean = 0, SD = 1), and fitness values were relativized by dividing individual fitness by the population mean. Selection opportunity was calculated as the variance in relative fitness. Selection differentials (from simple regressions) indicated the potential for trait change, while selection gradients (from multiple regressions) described the direction of selection (Wood and Brodie III 2016). Residuals were graphically examined for deviations from assumptions, and multicollinearity among traits was assessed using variance inflation factors (VIF) from the “car” package (Fox and Weisberg 2019). All analyses and graphs were performed in R version 4.5.1 (Windows OS) without an integrated development environment (IDE), using the packages: “boot” (Canty and Ripley 2024), “DescTools” (Signorell 2025), “emmeans” (Lenth 2020), “factoextra” (Kassambara and Mundt 2020), “FactoMineR” (Le et al. 2008), “ggplot2” (Wickham 2016), “lme4” (Bates et al. 2015), “reshape2” (Wickham 2007), and “vegan” (Oksanen et al. 2020).

## 3 Results

**FIGURE 3:**
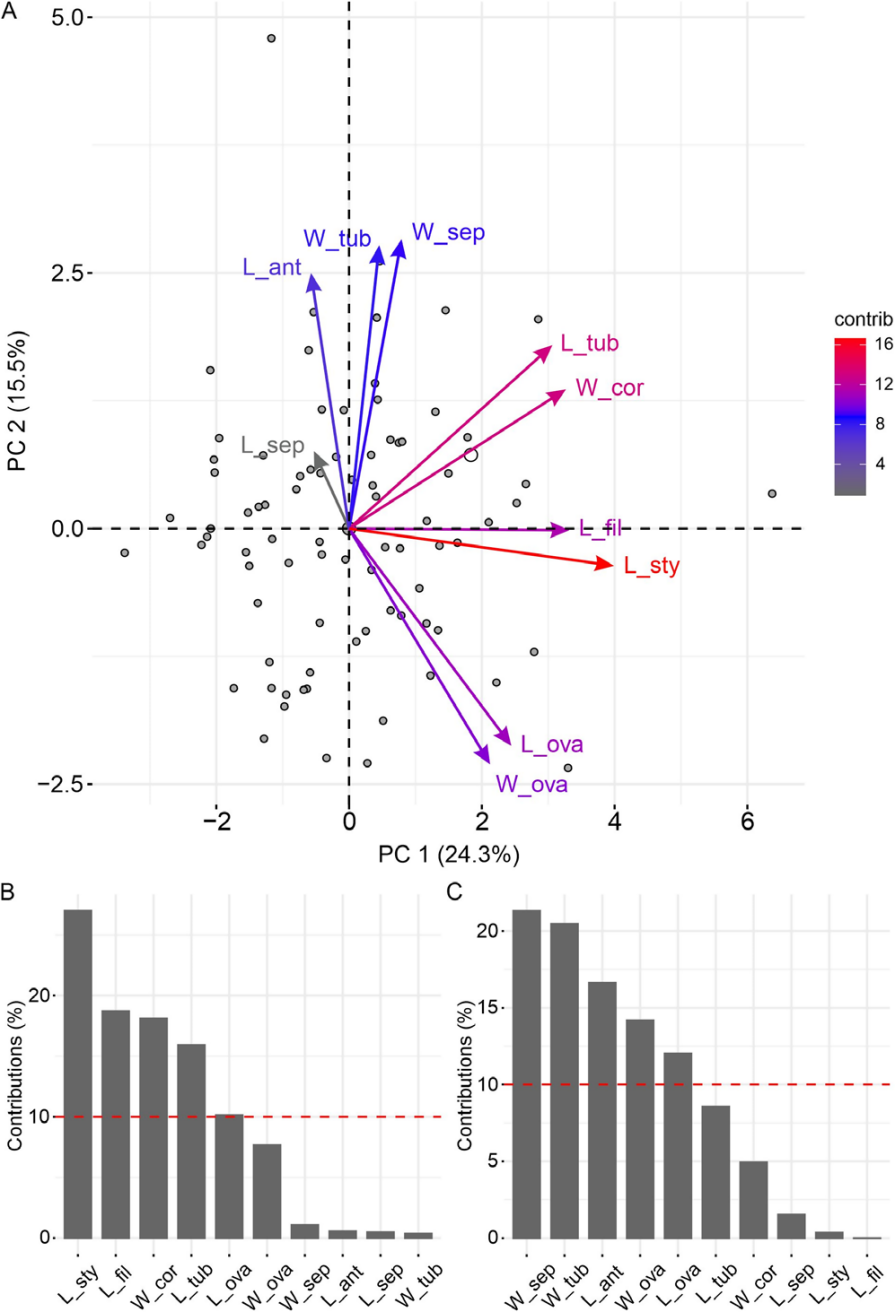
Principal component analysis (PCA) of standardized floral trait values in *Ipomoea cavalcantei*. (A) Distribution of individuals and floral traits in PCA space. (B) Variable contributions to axis 1. (C) Variable contributions to axis 2. L = length; W = width; sep = sepal; cor = corolla; tub = corolla tube; fil = filament; ant = anther; ova = ovary; sty = style.

**FIGURE 4:**
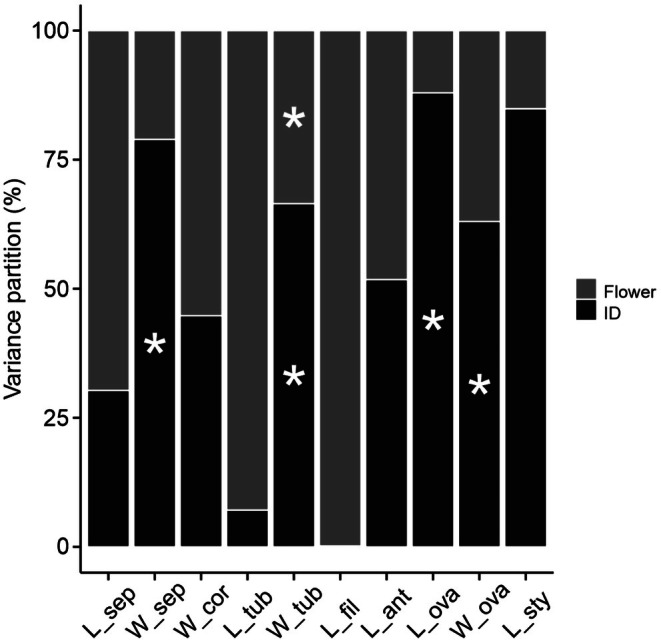
Variance partition analysis on floral traits in *Ipomoea cavalcantei*. Bars marked with an asterisk indicate significant variance components (*p* < 0.05). Black bars (ID) represent among‐individual variation; gray bar (Flower), within‐individual variation; L = length; W = width; sep = sepal; cor = corolla; tub = corolla tube; fil = filament; ant = anther; ova = ovary; sty = style.

The flower of *I. cavalcantei* has ovate sepals free from each other and a tubular corolla with epipetalous stamens. The reproductive whorls are exposed above the opening of the corolla tube, with stamens longer than the pistil (Table 1). Sepal and anther lengths showed the highest coefficients of variation, exceeding 20%, and did not exhibit intraspecific uniformity (Table 1). The first two principal components (PCs) explained 39.8% of the data variation, with individuals broadly distributed in the multivariate trait space, showing no clustering or gradient along these axes (Figure 3A). This distribution suggests uniformity in trait combinations across the sampled population. Style length contributed 27.1% of the variation in axis 1 (Figure 3B), while sepal width and corolla tube width contributed 21.3% and 20.4%, respectively, of the variation in axis 2 (Figure 3C), indicating greater contributions of these traits to phenotypic variation. In the variance partitioning analysis, sepal width, ovary length, and ovary width varied predominantly between individuals (> 63%), whereas corolla tube width varied both among individuals (66.5%) and among flowers of the same individual (33.5%; Figure 4).

**TABLE 1:** External floral morphometry and correlations among floral trait measurements in *Ipomoea cavalcantei*.

Floral trait	Mean (mm)	SD	CV (CI%)	PC1	PC2	L_sep	W_sep	W_cor	L_tub	W_tub	L_fil	L_ant	L_ova	W_ova	L_sty
L_sep	13.8	5.7	40.98 (34.8–49.9)	−0.11	0.15		−0.06, 0.26	0.02, 0.48	−0.26, 0.4	−0.13, 0.21	−0.32, −0.01	−0.14, 0.05	−0.08, 0.25	−0.15, 0.21	−0.25, 0.52
W_sep	6.4	0.4	5.47 (4.7–6.5)	0.16	0.57	0.09		0.01, 0.44	−0.17, 0.27	−0.05, 0.33	−0.11, 0.3	−0.02, 0.33	−0.16, 0.25	−0.40, 0.05	−0.2, 0.25
W_cor	32.8	1.8	5.58 (4.8–6.6)	0.66	0.28	0.08	**0.24**		0.15, 0.54	−0.09, 0.37	−0.04, 0.39	−0.26, 0.08	0.07, 0.47	−0.12, 0.38	0.26, 0.6
L_tub	30.9	2.0	6.52 (5.7–7.7)	0.62	0.36	−0.11	0.06	**0.36**		−0.01, 0.4	0.09, 0.54	−0.15, 0.23	−0.18, 0.24	−0.22, 0.24	0.24, 0.63
W_tub	6.7	0.6	8.74 (7.6–10.4)	0.09	0.56	0.10	0.14	0.16	**0.20**		−0.16, 0.25	0.09, 0.52	−0.35, 0.11	−0.17, 0.33	−0.28, 0.07
L_fil	29.8	1.7	5.8 (5.0–6.9)	0.67	−0.003	−0.14	0.12	**0.20**	**0.34**	0.03		−0.23, 0.18	−0.05, 0.34	0.05, 0.43	0.21, 0.71
L_ant	6.4	2.3	35.54 (30.3–43.2)	−0.12	0.51	−0.03	0.15	−0.01	0.03	0.13	−0.06		−0.54, −0.1	−0.37, −0.04	−0.33, 0.01
L_ova	1.4	0.2	11.2 (9.7–13.3)	0.50	−0.43	−0.01	0.06	**0.28**	0.02	−0.14	0.14	−0.14		0.23, 0.57	0.04, 0.47
W_ova	1.4	0.1	10.26 (8.9–12.2)	0.43	−0.47	0.004	**−0.19**	0.13	0.005	0.09	**0.24**	−0.10	**0.40**		−0.03, 0.43
L_sty	31.7	2.3	7.1 (6.1–8.4)	0.81	−0.07	−0.09	0.03	**0.44**	**0.46**	−0.11	**0.50**	−0.09	**0.28**	**0.22**	

*Note:* Bold values indicate significant Pearson correlation coefficients (empirical matrix). The lower triangle presents the Pearson correlation coefficients, and the upper triangle shows the respective confidence intervals (lower CI, upper CI).

Abbreviations: ant, anther; CI, confidence interval; cor, corolla; CV, coefficient of variation; fil, filament; L, length; ova, ovary; PC1, loading values of principal component axis 1; PC2, loading values of principal component axis 2; SD, standard deviation; sep, sepal; sty, style; tub, corolla tube; W, width.

Regarding trait relationships, 58% of the significant correlations were weak, reflecting limited covariation among most floral traits. Although Pearson's coefficients indicated weak to moderate associations, the confidence intervals showed variability in these patterns (Table 1). Style length had five significant correlations out of the nine possible, with weak to moderate associations with corolla width, corolla tube length, anther length, ovary length, and ovary width (Table 1). Modularity analysis revealed significant associations of weak magnitude for the morphological (*r* = 0.29, 95% CI: 0.37–0.85, *p* = 0.018) and developmental (*r* = 0.27, 95% CI: 0.21–0.75, *p* = 0.047) criteria. The association for the functional criterion was weaker and not significant (*r* = 0.21, 95% CI: 0.21–0.75, *p* = 0.089), indicating developmental constraints without functional integration. The Wagner‐Cheverud index was low (*I*_*c*_ = 0.11), suggesting weak phenotypic integration. This result points to loose trait co‐regulation, with the traits defined within modules not showing strong functional or developmental association.

**FIGURE 5:**
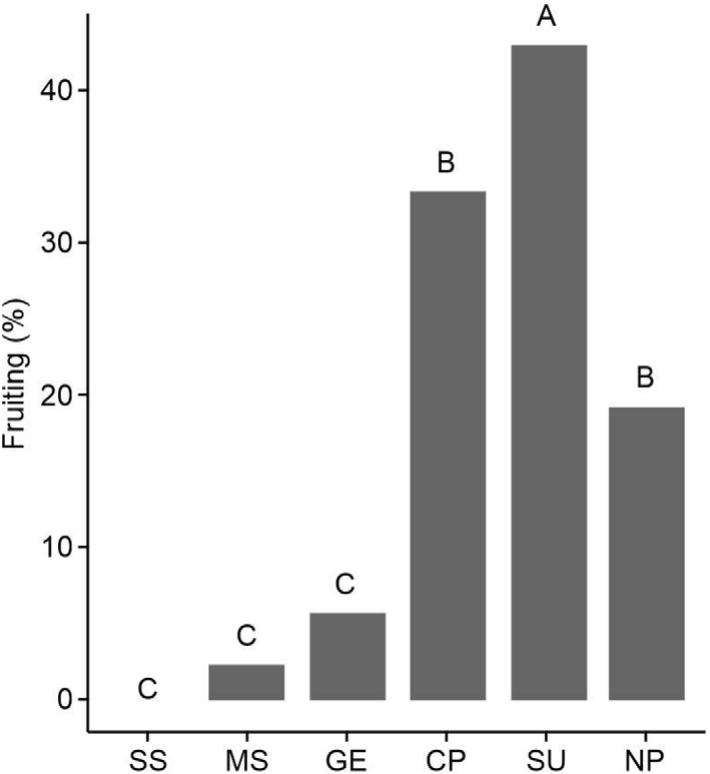
Results of pollination treatments in *Ipomoea cavalcantei*. Bars with the same letter indicate no significant differences among treatments (*p* > 0.05; *post hoc* Tukey test). SS = spontaneous self‐pollination; MS = manual self‐pollination; GE = geitonogamous pollination; CP = cross‐pollination; SU = pollen supplementation; NP = natural pollination.

In relation to the mating system, the full model explained more deviance than the null model (LRT: χ^2^ = 171.61, df = 5, *p* < 0.001), indicating differences in fruit set among pollination treatments. No fruits formed under spontaneous self‐pollination, and this result did not differ from manual self‐pollination (2.2%; estimate = −0.02, SE = 0.01, *t* = −1.56, *p* = 0.62) or geitonogamous pollination (5.6%; estimate = −0.05, SE = 0.02, *t* = −2.51, *p* = 0.12) (Figure 5). These three treatments differed from cross‐pollination (33.3%) (Figure 5, Table S4). There was no difference between natural pollination (19.1%) and cross‐pollination (estimate = −0.01, SE = 0.06, *t* = −0.18, *p* = 0.99; Figure 5). Pollen supplementation resulted in the highest fruiting rate (42.9%), differing from all other treatments (Figure 5, Table S4), suggesting pollen limitation. The proportion of fruits formed per plant (female fitness) was 0.15 ± 0.01, resulting in an opportunity for selection of 0.63 ± 0.08.

**FIGURE 6:**
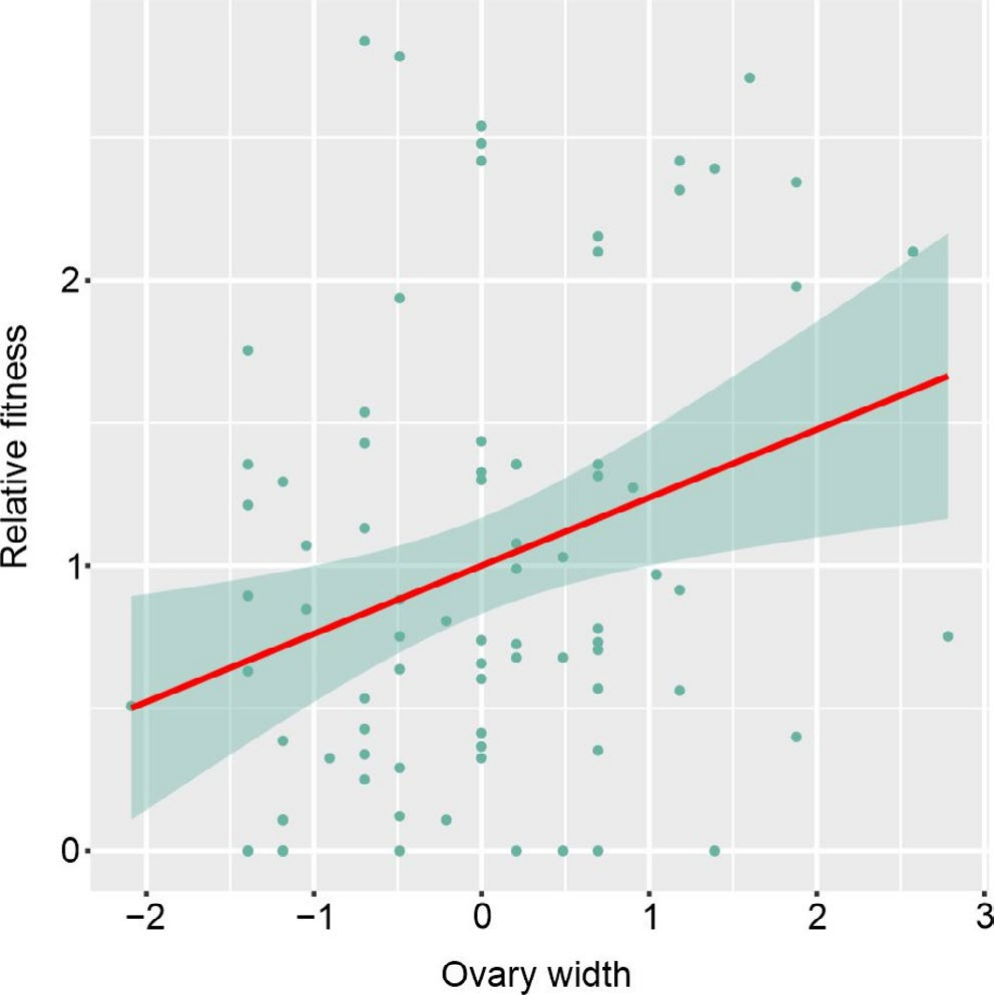
Positive directional selection on ovary width in the studied population of *Ipomoea cavalcantei*, based on the relationship between standardized ovary width and relative fitness.

In phenotypic selection analyses, the selection differential was significant only for ovary width in the linear model (Table 2). No traits had significant selection differentials in the nonlinear analysis (Table 2). Based on the variance partitioning results, corolla tube width, sepal width, and ovary width were included in the gradient selection analysis. Ovary length was excluded due to correlation with ovary width (*r* = 0.40; Table 1). There was no collinearity among the selected variables (VIF < 2). Only the ovary width showed a significant association in the linear model, and no significant associations were detected in the nonlinear or correlational models (Table 3). These findings indicate a positive directional change in floral phenotype: individuals with wider ovaries than the population average tend to exhibit greater relative fitness (Figure 6).

**TABLE 2:** Results of the selection differentials analysis for floral traits of *Ipomoea cavalcantei*.

Floral trait	*S*_*i*_ (SE)	*C*_*i*_ (SE)
L_sepal	0.0317 (0.0892)	0.006 (0.0105)
W_sepal	−0.1591 (0.0875)	0.0861 (0.0755)
W_corolla	0.0239 (0.0893)	0.0236 (0.0866)
L_tube	−0.1343 (0.088)	−0.0015 (0.0646)
L_tube	−0.0596 (0.089)	0.0938 (0.0615)
L_filament	0.0143 (0.0893)	−0.0188 (0.0289)
L_anther	−0.0357 (0.0889)	−0.0031 (0.0106)
L_ovary	0.1531 (0.0876)	0.012 (0.0459)
W_ovary	**0.2388 (0.0852)**	0.0583 (0.0646)
L_style	0.0522 (0.0891)	0.01 (0.0398)

*Note:* Significant *p‐*values (< 0.05) are in bold.

Abbreviations: *C*_*i*_, nonlinear selection coefficient; SE, standard error; *S*_*i*_, linear selection coefficient.

**TABLE 3:** Results of the selection gradients analysis for floral traits of *Ipomoea cavalcantei*.

Floral trait	*B*_*i*_ (SE)	*Y*_*ii*_ (SE)	*Y*_*ij*_ (SE)	
			W_tube	W_ovary
W_sepal	−0.1081 (0.0876)	0.1531 (0.1614)	0.015 (0.1122)	−0.0622 (0.0936)
W_tube	−0.0646 (0.0864)	0.2365 (0.1408)		−0.0519 (0.0912)
W_ovary	**0.2245 (0.0871)**	−0.0578 (0.157)		

*Note:* Significant *p*‐values (< 0.05) are in bold.

Abbreviations: *B*_*i*_, linear selection coefficient; SE, standard error; *Y*_*ii*_, nonlinear selection coefficient; *Y*_*ij*_, correlational selection coefficient.

## 4 Discussion

We found that individuals of *I. cavalcantei* were dispersed in phenotypic space, with weak covariances and low floral integration across most studied traits. Intraspecific and intraindividual contributions to variation differed among floral traits. Despite this, *I. cavalcantei* depends on pollinators for sexual reproduction and shows indications of self‐incompatibility, with phenotypic selection detected only for ovary width.

### 4.1 Floral Integration

The weak correlations among floral traits in *I. cavalcantei* are consistent with expectations for taxa with generalist pollination systems, where pollinators differ in form and impose selection from various directions (Berg 1960). Nonetheless, trait‐specific integration was evident, particularly in the number of correlations involving style length. This trait showed among the highest interindividual variation and was associated with both attraction/access traits (corolla) and pollen transfer traits (stamen). In *Ipomoea*, combinations of traits involved in pollen transfer likely respond more to pollinator‐driven selection than traits related to visitor attraction (Rosas‐Guerrero et al. 2011). Style length has been previously recognized as central to floral integration and pollen transfer efficiency, responding to the identity of floral visitors (Sosenski et al. 2010; Pérez‐Barrales et al. 2014). In *I. cavalcantei*, the markedly exserted styles are adapted to hummingbird pollination (Babiychuk et al. 2019).

The observed low floral integration and widespread distribution of individuals in trait space, coupled with limited trait covariation, align with patterns found in other generalist and self‐incompatible *Ipomoea* species (Rosas‐Guerrero et al. 2011; Fornoni et al. 2016; but see Delgado‐Dávila et al. 2016). The positive correlations among the length and width of most floral structures suggest the maintenance of larger flowers in the population, a trait likely contributing to pollinator attraction alongside the reddish coloration (Babiychuk et al. 2019). Additionally, floral UV reflectance (unpublished data) may attract bees (Lunau et al. 2011), reinforcing the generalist nature of the pollination system.

As only the morphological and developmental modules were significant, the basic floral structure (bauplan) is likely conserved, with no requirement for new interactions between floral whorls to accommodate pollinator morphology. Developmental modularity may promote evolvability by allowing some components to respond to selection independently (Klingenberg 2008; Wagner et al. 2007), supporting adaptation to changes in pollinator assemblages. In scenarios of pollinator shifts due to habitat disturbance or climate change, modularity may permit trait‐specific adjustments without altering overall floral architecture (Armbruster et al. 2014; Gómez, Perfectti, and Klingenberg 2014). This flexibility can benefit generalist species by enabling phenotypic responses to changing selection regimes while retaining core developmental patterns (Pigliucci 2008; Pavlicev and Wagner 2012).

In contrast, our results diverge from those reported in studies of phenotypic integration in endemic species. High floral integration has been proposed to constrain evolutionary responses, as trait changes may be correlated (Schlichting 1989). Endemic species tend to exhibit strong integration, possibly contributing to their restricted distributions and habitat specialization (Callahan and Waller 2000; Hermant et al. 2013). However, Hermant et al. (2013) focused on vegetative morphological traits and flowering time, suggesting the possibility of a decoupling between vegetative and reproductive integration within species (Armbruster et al. 1999; Brock and Weinig 2007). This decoupling is not necessarily expected in generalist‐pollinated species (Berg 1960), highlighting the need to examine both vegetative and reproductive traits in future studies of phenotypic integration in endemic plants.

### 4.2 Mating System

Patterns in floral traits of *I. cavalcantei* suggest selective pressures associated with pollen transfer efficiency, raising questions about its mating dynamics and potential reproductive constraints. Pollination experiments showed that *I. cavalcantei* depends on biotic pollinators for sexual reproduction, although fruit set was low in self‐pollination and geitonogamy treatments. At least nine hummingbird species and two long‐tongued bees have been observed visiting its flowers (Babiychuk et al. 2019) and are likely pollinators. The results also indicate the presence of a genetic self‐incompatibility system. While self‐compatibility is often reported in the family (Hassa et al. 2023), self‐incompatibility has been documented in other *Ipomoea* species (McDonald et al. 2011; Delgado‐Dávila et al. 2016).

Although the species is predominantly xenogamous, the studied population exhibits pollen limitation, possibly linked to changes in habitat conditions and the species' narrow and localized distribution (Lanes et al. 2018; Rodrigues et al. 2020), which may affect plant‐pollinator interactions. Pollen limitation is more commonly observed in self‐incompatible and endemic species than in self‐compatible or widespread species (Knight et al. 2005; Alonso et al. 2010). It may result from reduced pollen deposition on the stigma due to scarce or ineffective pollinators (Alonso et al. 2013). Pollen quality may also contribute, as incompatible or self‐pollen on stigmas can reduce fruit set (Alonso et al. 2013; Li et al. 2020). In *I. cavalcantei*, pollen quality may be the more limiting factor, based on three lines of evidence. (1) The species receives visits from a diverse assemblage of floral visitors (Babiychuk et al. 2019), suggesting that pollinator quantity is not the main limiting factor. (2) Pollinator behavior varies. Hummingbirds often promote geitonogamous pollination (pers. obs.), which may result in fertilization failure due to self‐incompatibility or physical inhibition, such as pollen clogging (Wilcock and Neiland 2002). Bees may contribute more to long‐distance pollen transfer among individuals (Schmidt‐Lebuhn et al. 2019). (3) The pollen supplementation treatment yielded greater fruit set than cross‐pollination (Figure 5), suggesting that heterogeneous pollen loads enhance reproductive success (Dener et al. 2021). Self‐incompatible endemic species like *I. cavalcantei*, particularly in species‐rich environments, may face increased risk of pollination failure, reinforcing their priority in conservation planning (Alonso et al. 2010).

### 4.3 Phenotypic Selection

The reproductive dynamics observed here, particularly those affecting fruit set, raise the question of whether floral morphology is under current selective pressure. In our analysis of phenotypic selection on the floral traits of *I. cavalcantei*, only one trait exhibited a significant selection pattern. Directional selection on ovary width, as detected in this study, is seldom reported in angiosperms, possibly because this trait is often excluded from phenotypic selection analyses (Schmid 1992; Caruso et al. 2019). A likely explanation for this pattern involves seed production. Ovary locule size is related to the number and arrangement of ovules (Silva et al. 2020), which can influence seed size and associated structures, such as the long trichomes on mature seeds of *I. cavalcantei* (Simão‐Bianchini et al. 2016). Although no variation in seed number across pollination treatments was recorded, consistent with findings by Delgado‐Dávila et al. (2016) in four *Ipomoea* species, we observed in‐field variation in fruit shape and in the number of seeds per fruit among individuals. The ovoid capsules (Simão‐Bianchini et al. 2016) tend to be narrower when containing fewer than four seeds, likely due to seed abortion, and occur less frequently in the population (unpublished data), supporting the hypothesis that ovary width is associated with seed number.

Producing fewer seeds per fruit might result in seedlings with greater vigor, assuming that increased resource availability allows for larger or heavier seeds (Garwood 1983; Khurana and Singh 2001). However, the high rate of pre‐dispersal seed predation (Santos et al. 2023) reduces the number of viable seeds and may affect fitness (Kolb et al. 2007; Stachurska‐Swakón et al. 2018). Under such circumstances, increased seed output may raise the likelihood of successful reproduction. Seed predators, therefore, may act as selective agents (Kolb et al. 2007; Kolb and Ehrlén 2010; Sun et al. 2016), shaping the maintenance of ovary width as a heritable trait through effects on female fitness (viable seed number).

No significant selection was detected in corolla dimensions, contrary to expectations for hummingbird‐pollinated species (Campbell et al. 1991; Nattero and Cocucci 2007; Faure et al. 2022). This outcome is notable given that hummingbird pollination was identified as a key step in the evolutionary differentiation of *I. cavalcantei* from its sister species *I. marabaensis* (Babiychuk et al. 2017, 2019). Variation in corolla tube width, with broader flowers recorded on the N1 plateau, was also documented by Babiychuk et al. (2019). Such variation may reflect morphological matching and pollinator filtering (Muchhala 2007; Babiychuk et al. 2019). Nonetheless, neither selection differentials nor gradients were significant for this trait, possibly due to the composition of the floral visitor assemblage. Differences in resource use among hummingbird species with distinct behaviors can influence visitation patterns (Hadley et al. 2017; Torres‐Vanegas et al. 2019; Wanderley et al. 2020) and, in turn, the selective pressures they impose. Multiple hummingbird and bee species have been observed visiting *I. cavalcantei* flowers (Babiychuk et al. 2019). In mixed‐pollination systems, divergent visitor preferences can weaken or obscure selection on floral traits (Gómez and Zamora 2006). For instance, hummingbirds and bees may favor different corolla traits, resulting in diffuse or opposing selection, as reported for other species (Sahli and Conner 2011; Strelin et al. 2017). In addition, shifts in pollinator assemblages across space or time may lead to inconsistent selection pressures across populations or years (Gómez et al. 2008; Caruso et al. 2019).

Aside from pollinators, antagonists can also influence phenotypic selection (Strauss and Whittall 2006; Gélvez‐Zúñiga et al. 2018), as suggested by the presence of nectar robbers on *I. cavalcantei* flowers (Babiychuk et al. 2019). These floral larcenists often exploit nectar from long‐corolla flowers (Lara and Ornelas 2001; Lázaro et al. 2015) and may alter pollinator foraging behavior (Yanwen et al. 2007; Irwin et al. 2010). Hummingbirds tend to avoid nectar‐depleted flowers, whereas some bees may be less discriminating (Irwin et al. 2010). Thus, nectar robbers could affect pollinator visitation rates and modify the selection landscape on the corolla. Even when not currently under selection, such traits can still display heritable variation. This suggests that, despite the absence of adaptive change in the present context, genetic variation remains in the population and may respond to future selection (Castellanos et al. 2023).

## 5 Conclusions

*Ipomoea cavalcantei* is self‐incompatible and relies on biotic pollinators for reproduction, exhibiting pollen limitation. The correlations involving style length are likely associated with selective pressures from pollinators on traits influencing pollen deposition. In addition, ovary width varied among flowers and was under positive directional selection, with pre‐dispersal seed predators likely acting as the selective agents. These findings suggest a more complex relationship between floral traits and pollinator‐mediated selection than commonly assumed for hummingbird‐pollinated species, as the traits under selection were not those typically linked to mechanical fit (e.g., corolla size). In this species, pollinators contribute to maximizing seed production, which may help ensure reproductive success despite seed predation. This could inform the identification of target individuals for use as seed sources in in situ conservation. However, given the absence of strong selection on most floral traits, floral phenotype alone may not be a reliable criterion for such selection. Instead, the evolutionary potential of the population is likely tied to the maintenance of high phenotypic diversity through the conservation of randomly selected individuals. This is particularly relevant for *I. cavalcantei*, a rare, endemic, and self‐incompatible species subject to pollen limitation. These reproductive traits may increase vulnerability to environmental change and demographic shifts. Conservation strategies should therefore emphasize the preservation of both genetic and phenotypic diversity to maintain compatibility among individuals and potential for adaptation. Understanding factors related to floral integration and phenotypic selection informs both the species' natural history and the design of effective in situ conservation measures.

## Author Contributions

**Adriano Valentin‐Silva:** data curation (equal), formal analysis (equal), methodology (equal), software (equal), validation (equal), visualization (equal), writing – original draft (lead). **Ana Carolina Galindo da Costa:** conceptualization (equal), data curation (equal), investigation (equal), methodology (equal), writing – review and editing (equal). **Arthur Domingos‐Melo:** conceptualization (equal), formal analysis (equal), methodology (equal), validation (equal), writing – review and editing (equal). **Lucas Erickson Nascimento da Costa:** investigation (equal), writing – review and editing (equal). **Kleber Resende Silva:** investigation (equal), writing – review and editing (equal). **Carolina da Silva Carvalho:** funding acquisition (equal), resources (equal), writing – review and editing (equal). **Maurício Takashi Coutinho Watanabe:** conceptualization (equal), funding acquisition (equal), investigation (equal), project administration (lead), resources (equal), supervision (lead), writing – review and editing (equal).

## Conflicts of Interest

Ana Carolina Galindo da Costa, Carolina da Silva Carvalho, and Maurício Takashi Coutinho Watanabe work (Or Worked) for a non‐profit research institution funded by the private mining company Vale S.A. Adriano Valentin‐Silva, Lucas Erickson Nascimento da Costa, and Kleber Resende Silva receive fellowships from a project financed by Vale S.A.

## Supporting information


**Table S1:** Matrix based on morphological criteria. Colors indicate floral whorls: gray = calyx; blue = corolla; pink = androecium; green = gynoecium. L = length; W = width; sep = sepal; cor = corolla; tub = corolla tube; fil = filament; ant = anther; ova = ovary; sty = style.
**TABLE S2:** Matrix based on functional criteria. Colors indicate functional modules: gray = visual attraction; blue = pollen export; pink = pollen reception. L = length; W = width; sep = sepal; cor = corolla; tub = corolla tube; fil = filament; ant = anther; ova = ovary; sty = style.
**TABLE S3:** Matrix based on developmental criteria. Colors indicate developmental modules: gray = sepals; blue = epipetalous stamens; pink = and the pistil. L = length; W = width; sep = sepal; cor = corolla; tub = corolla tube; fil = filament; ant = anther; ova = ovary; sty = style.
**TABLE S4:** Results of comparisons between pollination treatments performed on *Ipomoea cavalcantei* using *post hoc* Tukey tests. SS = spontaneous self‐pollination; MS = manual self‐pollination; GE = geitonogamous pollination; CP = cross‐pollination; SU = pollen supplementation; NP = natural pollination; SE = standard error.

## Data Availability

The datasets presented in the study are included in the article/Supporting Information. Further inquiries can be directed to the corresponding author.
