# Supplementary material for: Floral Integration and Phenotypic Selection on Floral Traits of Ipomoea cavalcantei (Convolvulaceae), a Rare, Endemic, and Endangered Species to the Amazon Ironstone Outcrops
Source: Ecol Evol. 2025 Sep 13;15(9):e72133. doi: 10.1002/ece3.72133 (PMC12432336; doi:10.1002/ece3.72133)
Supplement: Supplementary file 1 — Table S1: Matrix based on morphological criteria. Colors indicate floral whorls: gray = calyx; blue = corolla; pink = androecium; green = gynoecium. L = length; W = width; sep = sepal; cor = corolla; tub = corolla tube; fil = filament; ant = anther; ova = ovary; sty = style. TABLE S2: Matrix based on functional criteria. Colors indicate functional modules: gray = visual attraction; blue = pollen export; pink = pollen reception. L = length; W = width; sep = sepal; cor = corolla; tub = corolla tube; fil = filament; ant = anther; ova = ovary; sty = style. TABLE S3: Matrix based on developmental criteria. Colors indicate developmental modules: gray = sepals; blue = epipetalous stamens; pink = and the pistil. L = length; W = width; sep = sepal; cor = corolla; tub = corolla tube; fil = filament; ant = anther; ova = ovary; sty = style. TABLE S4: Results of comparisons between pollination treatments performed on Ipomoea cavalcantei using post hoc Tukey tests. SS = spontaneous self‐pollination; MS = manual self‐pollination; GE = geitonogamous pollination; CP = cross‐pollination; SU = pollen supplementation; NP = natural pollination; SE = standard error. [file ECE3-15-e72133-s001.docx]

Supplementary Table 1: Matrix based on morphological criteria. Colors indicate floral whorls: gray = calyx; blue = corolla; pink = androecium; green = gynoecium. L = length; W = width; sep = sepal; cor = corolla; tub = corolla tube; fil = filament; ant = anther; ova = ovary; sty = style.

|  | L_sep | W_sep | W_cor | L_tub | W_tub | L_fil | L_ant | L_ova | W_ova | L_sty |
| --- | --- | --- | --- | --- | --- | --- | --- | --- | --- | --- |
| L_sep | 1 | 1 | 0 | 0 | 0 | 0 | 0 | 0 | 0 | 0 |
| W_sep | 1 | 1 | 0 | 0 | 0 | 0 | 0 | 0 | 0 | 0 |
| W_cor | 0 | 0 | 1 | 1 | 1 | 0 | 0 | 0 | 0 | 0 |
| L_tub | 0 | 0 | 1 | 1 | 1 | 0 | 0 | 0 | 0 | 0 |
| W_tub | 0 | 0 | 1 | 1 | 1 | 0 | 0 | 0 | 0 | 0 |
| L_fil | 0 | 0 | 0 | 0 | 0 | 1 | 1 | 0 | 0 | 0 |
| L_ant | 0 | 0 | 0 | 0 | 0 | 1 | 1 | 0 | 0 | 0 |
| L_ova | 0 | 0 | 0 | 0 | 0 | 0 | 0 | 1 | 1 | 1 |
| W_ova | 0 | 0 | 0 | 0 | 0 | 0 | 0 | 1 | 1 | 1 |
| L_sty | 0 | 0 | 0 | 0 | 0 | 0 | 0 | 1 | 1 | 1 |

Supplementary Table 2: Matrix based on functional criteria. Colors indicate functional modules: gray = visual attraction; blue = pollen export; pink = pollen reception. L = length; W = width; sep = sepal; cor = corolla; tub = corolla tube; fil = filament; ant = anther; ova = ovary; sty = style.

|  | L_sep | W_sep | W_cor | L_tub | W_tub | L_fil | L_ant | L_ova | W_ova | L_sty |
| --- | --- | --- | --- | --- | --- | --- | --- | --- | --- | --- |
| L_sep | 1 | 1 | 1 | 1 | 1 | 0 | 0 | 0 | 0 | 0 |
| W_sep | 1 | 1 | 1 | 1 | 1 | 0 | 0 | 0 | 0 | 0 |
| W_cor | 1 | 1 | 1 | 1 | 1 | 0 | 0 | 0 | 0 | 0 |
| L_tub | 1 | 1 | 1 | 1 | 1 | 0 | 0 | 0 | 0 | 0 |
| W_tub | 1 | 1 | 1 | 1 | 1 | 0 | 0 | 0 | 0 | 0 |
| L_fil | 0 | 0 | 0 | 0 | 0 | 1 | 1 | 0 | 0 | 0 |
| L_ant | 0 | 0 | 0 | 0 | 0 | 1 | 1 | 0 | 0 | 0 |
| L_ova | 0 | 0 | 0 | 0 | 0 | 0 | 0 | 1 | 1 | 1 |
| W_ova | 0 | 0 | 0 | 0 | 0 | 0 | 0 | 1 | 1 | 1 |
| L_sty | 0 | 0 | 0 | 0 | 0 | 0 | 0 | 1 | 1 | 1 |

Supplementary Table 3: Matrix based on developmental criteria. Colors indicate developmental modules: gray = sepals; blue = epipetalous stamens; pink = and the pistil. L = length; W = width; sep = sepal; cor = corolla; tub = corolla tube; fil = filament; ant = anther; ova = ovary; sty = style.

|  | L_sep | W_sep | W_cor | L_tub | W_tub | L_fil | L_ant | L_ova | W_ova | L_sty |
| --- | --- | --- | --- | --- | --- | --- | --- | --- | --- | --- |
| L_sep | 1 | 1 | 0 | 0 | 0 | 0 | 0 | 0 | 0 | 0 |
| W_sep | 1 | 1 | 0 | 0 | 0 | 0 | 0 | 0 | 0 | 0 |
| W_cor | 0 | 0 | 1 | 1 | 1 | 1 | 1 | 0 | 0 | 0 |
| L_tub | 0 | 0 | 1 | 1 | 1 | 1 | 1 | 0 | 0 | 0 |
| W_tub | 0 | 0 | 1 | 1 | 1 | 1 | 1 | 0 | 0 | 0 |
| L_fil | 0 | 0 | 1 | 1 | 1 | 1 | 1 | 0 | 0 | 0 |
| L_ant | 0 | 0 | 1 | 1 | 1 | 1 | 1 | 0 | 0 | 0 |
| L_ova | 0 | 0 | 0 | 0 | 0 | 0 | 0 | 1 | 1 | 1 |
| W_ova | 0 | 0 | 0 | 0 | 0 | 0 | 0 | 1 | 1 | 1 |
| L_sty | 0 | 0 | 0 | 0 | 0 | 0 | 0 | 1 | 1 | 1 |

Supplementary Table 4: Results of comparisons between pollination treatments performed on *Ipomoea cavalcantei* using *post-hoc* Tukey tests. SS = spontaneous self-pollination; MS = manual self-pollination; GE = geitonogamous pollination; CP = cross-pollination; SU = pollen supplementation; NP = natural pollination; SE = standard error.

| Contrast | Estimate | SE | *t* | *p* |
| --- | --- | --- | --- | --- |
| SS - MS | -0.02 | 0.01 | -1.56 | 0.62 |
| SS - GE | -0.06 | 0.02 | -2.51 | 0.12 |
| SS - CP | -0.33 | 0.05 | -7.31 | < 0.0001 |
| SS - SU | -0.63 | 0.05 | -13.2 | < 0.0001 |
| SS - NP | -0.32 | 0.05 | -7.13 | < 0.0001 |
| MS - GE | -0.03 | 0.03 | -1.27 | 0.8 |
| MS - CP | -0.31 | 0.05 | -6.51 | < 0.0001 |
| MS - SU | -0.6 | 0.05 | -12.19 | < 0.0001 |
| MS - NP | -0.3 | 0.05 | -6.33 | < 0.0001 |
| GE - CP | -0.28 | 0.05 | -5.48 | < 0.0001 |
| GE - SU | -0.57 | 0.05 | -10.89 | < 0.0001 |
| GE - NP | -0.27 | 0.05 | -5.3 | < 0.0001 |
| CP - SU | -0.29 | 0.07 | -4.43 | 0.0001 |
| CP - NP | -0.01 | 0.06 | -0.17 | 0.99 |
| SU - NP | -0.3 | 0.07 | -4.62 | 0.0001 |
